# The American Rhinological Association

**Published:** 1889-08

**Authors:** R. S. Knode

**Affiliations:** Sec., Omaha, Neb. per A


THE AMERICAN RHINOLOGICAL ASSOCIATION will hold its Seventh Annual meeting at Chicago, Ill., August 28, 29 and 30, 1889.

The Committee on the examination of the inmates of insane asylums "will make their report" on the relation of Rhinal inflammations, to mind affections at this session.

R. S. KNODE, SEC.,
Omaha, Neb. per A.

